# Editorial: Chromosome bi-orientation, tension and the spindle-assembly checkpoint

**DOI:** 10.3389/fcell.2023.1245368

**Published:** 2023-07-04

**Authors:** Scott C. Schuyler

**Affiliations:** ^1^ Department of Biomedical Sciences, College of Medicine, Chang Gung University, Taoyuan, Taiwan; ^2^ Division of Head and Neck Surgery, Department of Otolaryngology, Chang Gung Memorial Hospital, Taoyuan, Taiwan

**Keywords:** mitosis, chromosomes, aurora kinase, tension, spindle checkpoint

I am very pleased to report on a Research Topic of two primary Research Articles and two Reviews published here as part of our Research Topic focused on advances and ideas in connection with chromosome bi-orientation, tension, and the spindle-assembly checkpoint. In setting out to collect articles for this Research Topic our goal was to attract manuscripts that present a wide variety of views and explore novel or under-represented research areas and ideas that we think are of importance in the field. We were pleased to have had the opportunity to participate in the publishing of such good quality articles towards achieving this goal.

Lee and Biggins reports on the discovery of a transcriptional regulatory network response after the loss of microtubule integrity—an area of critical importance, but one that we think is under-explored within the field. Notably, they have observed a Research Topic of genes that are downregulated in response to loss of microtubules within the Regulation of Ace2 Morphogenesis (RAM) pathway involved in, in part, the regulation of cytokinesis. Their article has established that proper regulation of mitotic transcriptional programs in response to a loss in microtubule integrality is a critical part of proper cell cycle progression. We look forward to future developments in this field of research especially in human cells and in the context of human diseases, and in connection with the application of anti-microtubule drugs targeting disease such as cancer.

Britigan et al., is focused on reporting the consequences of the over expression of the core mitotic regulator protein kinase Aurora B, a common condition within cancer cells. With compelling experiments from several different biological systems and by investigating multiple substrates, the authors report the unexpected result that over expression of Aurora B leads to a *decrease* in target substrate phosphorylation levels and kinase activity. They establish the mechanism as the mis-localization of the kinase due to an imbalance in binding partner stoichiometry. This important report, which has already been cited since publication, has an impact regarding the pursuit and development of Aurora B kinase inhibitors targeting cancer and why pre-screening potential patients to enroll in clinical trials for Aurora B levels first may be critically important.

Bunning and Gupta focused on the critical process of developing chromosomal tension for accurate chromosome segregation. Their writing provides the field with an up-to-date summary of our knowledge and, importantly, also highlights what we do not know and raises a series of critical unanswered questions that are going to shape the focus of the research in our field for decades to come. Evidence for the impact of the quality of their writing is provided by the fact the Review has already been cited since its publication earlier this year.

Ali and Stukenberg focused on the Aurora kinases and the fundamental role they play in spatial control in mitosis. The Aurora kinases are a highly conserved and ancient unique branch amongst the greater eukaryotic kinases that function at the heart of mitosis creating the ability of cells to execute accurate chromosome segregation. They highlight the functions of an Aurora B kinase gradient centered at the metaphase plate and Aurora A kinase gradients emanating from the spindle poles as geometric ‘beacons’ for chromosomes and molecules involved in regulating and executing chromosome segregation. The Review highlights the critical need to explore the concept of regulatory gradients giving rise to fine-scale geometric information within cells, not only in mitosis, but as a critical concept within the broader context of cell biology.

This Research Topic of four papers represents only a small portion of our larger field. Nonetheless, we hope the readers find these articles and reviews interesting, informative, and useful on the specific topic of chromosome bi-orientation, tension, and the spindle-assembly checkpoint. This ancient and fundamental element of eukaryotic cell biology is not only essential for life but, as all the papers have emphasized, a clear understanding of these biological processes is critical for having an informed understanding as to how we might better attack human diseases such as cancer. I wish to thank all the authors for their hard work in contributing to our Research Topic. I find this area of research to be very interesting and fascinating, and I anticipate many new discoveries and ideas in the field for decades to come.

